# Metabolic syndrome and its components are associated with thyroid volume in adolescents

**DOI:** 10.1186/s12902-021-00833-3

**Published:** 2021-08-28

**Authors:** Yang Xiao, Jingjing Mao, Xiaodong Mao, Qifeng Wang, Xingjia Li, Guofang Chen, Ling Guo, Huaying Huang, Yiming Mu, Shuhang Xu, Chao Liu

**Affiliations:** 1 Endocrine and Diabetes Center, Affiliated Hospital of Integrated Traditional Chinese and Western Medicine, Nanjing University of Chinese Medicine (Jiangsu Province Hospital on Integration of Chinese and Western Medicine), Nanjing, China; 2 Department of Endocrinology, Wujin Hospital of Traditional Chinese Medicine, Changzhou, China; 3 Department of Endocrinology, Chinese PLA General Hospital, Beijing, China

**Keywords:** Thyroid volume, Metabolic syndrome, Adolescents

## Abstract

**Objective:**

To explore the association between metabolic syndrome (MetS) and its component and thyroid volume in Chinese adolescents, and to compare the detection rate of MetS under the three different diagnostic criteria.

**Methods:**

A total of 1097 school students (610 males and 487 females, ages 12–15 years) were enrolled. All the participants underwent physical examination, biochemical test, and thyroid gland ultrasonography. The thyroid volume of normal, overweight and obese group was compared. We also analyzed the association between the number of MetS components and thyroid volume. Linear and multiple linear regression were applied to explore the association between metabolic parameters and thyroid volume.

**Results:**

The thyroid volume of the males in overweight (*t* = 3.784, *P* < 0.001) and obese group (*t* = 5.068, *P* < 0.001) was significantly larger than that in normal group; the thyroid volume of the females in overweight group (*t* = 4.627,*P* < 0.001) was significantly larger than that of normal group. As the number of MetS components increased, the thyroid volume also increased significantly (*F* = 10.64, *P* < 0.01). Height, weight, body mass index (BMI), waist circumference, hip circumference, systolic blood pressure, fasting insulin, homeostasis model assessment of insulin resistance (HOMA-IR), uric acid and triglyceride were all positively associated with thyroid volume in the adolescents (*P* all < 0.001). Meanwhile, there was a negative association between high-density lipoprotein cholesterol (HDL-C) and thyroid volume (*P* < 0.001). According to multiple linear regression, waist circumference (*β* = 0.029, 95 %CI: 0.015 ~ 0.042; *P* < 0.01) and waist height ratio (*β* = 3.317, 95 %CI: 1.661 ~ 4.973; *P* < 0.01) were predict factors of thyroid volume. No statistical difference was found in the detection rates of metabolic syndrome under the three diagnostic criteria.

**Conclusions:**

Overweight, obesity and metabolic syndrome was associated with adolescent thyroid volume. Central obesity may be an independent risk factor for thyroid enlargement in adolescents.

## Introduction

The prevalence of goiter in adult has decreased from 22.80 % to 1996 when universal salt iodization program was implemented to the 5.02 % in 2016 in mainland China [1]. However, goiter is still common in adolescents, which may be attributable to multiple factors such as genetic predisposition, autoimmune disorders, puberty-related factors, and other factors [2]. Recently, the association of metabolic disorders with thyroid functional/morphological abnormalities is becoming an intriguing area of research in thyroidology. Previously we found a positive association between Metabolic Syndrome (MetS) and formation of thyroid nodules in adult [3]. Among multiple components of MetS, increased waist circumference (WC) might increase the prevalence of thyroid nodules [3, 4]. At the same time, metabolic disorders has been found to be closely related to thyroid volume in adults in recent years [5–7]. These findings call into question whether MetS is associated with thyroid morphological change in adolescents. A clear understanding of this association is helpful for the control of Metabolic syndrome (MetS) and prevention of goiter in adolescents.

MetS is a series of interrelated physiological, biochemical, clinical and metabolic manifestations, including obesity, dyslipidemia, hypertension, impaired glucose regulation, etc. The prevalence of MetS in both adult and adolescents has also been increasing in recent years [8, 9]. Unfortunately, the diagnostic criteria of MetS in children remain controversial [10]. There are 3 most widely used MetS diagnostic criteria for children and adolescents in China [11–13]. The discrepancies among these criteria exist and result in different detection rates of MetS in adolescents [14], which further affects the policy-making in MetS intervention in children and adolescents.

Therefore, we undertook the present study to clarify whether MetS and its components are significantly associated with thyroid volume in Chinese adolescents. Meanwhile, we compared the detection rate of MetS under the three different diagnostic criteria.

## Subjects and methods

### Participants

This study was part of a larger study designed to address the effects of genetic factors on incidences of type 2 diabetes among Chinese adolescents in multiple centers. The study was approved by the ethics committees in Chinese PLA General Hospital. It was carried out in Changzhou City, Jiangsu Province as a subcenter. A total of 1 097 students aged 12 to 15 years were sampled from three local junior high schools from October 1, 2017 to November 30, 2017. All the participants underwent physical examination and thyroid ultrasound examination. Blood samples were collected. Finally, 834 participants completed the questionnaire survey. All participants were personally interviewed by trained interviewers who recorded their demographic information, clinical and family history, smoking and dietary habits. The participants under hypoglycemic, antihypertensive, and/or lipid lowering treatment were also included in the study and all these treatments were also recorded. In accordance with the guidelines of the Declaration of Helsinki, all the participants and their guardians provided informed consent.

### Questionnaire and physical examination

Data were collected at physical examination centers of local medical institutions. During the visit, a trained researcher asked the interviewee to complete a standard questionnaire in Chinese, pertaining to age, gender, history and life habits. The height, weight, waist circumference, hip circumference, heart rate, systolic and diastolic blood pressure were noted. All the subjects were asked not to drink any beverage, with the exception of water, and take heavy exercise at least 1 h before physical examination and blood collection. Body weight and height were measured twice during the examination. Weight (precision of kilogram) and height (precision centimeter) were measured with the subjects wearing light clothing and no shoes. Body mass index (BMI) was calculated as weight divided by height squared.

### Laboratory test and thyroid ultrasound

After 8 h of fasting, 5 ml of venous blood sample was drawn from each participant in 8:00 ~ 9:00 am. After centrifugation, fasting blood glucose (FBG) was detected with glucose oxidase method. Total cholesterol (TC), high-density lipoprotein cholesterol (HDL-C), low-density lipoprotein cholesterol (LDL-C), uric acid (UA) and other biochemical indexes were detected by enzymatic method (Roche Diagnostics GmbH, C8000 Biochemistry analyzer, Switzerland). Fasting insulin was detected by chemiluminescence (Roche Diagnostics GmbH, E601 Automatic electrochemical luminescence analyzer, Switzerland). Homeostasis model assessment of insulin resistance (HOMA-IR) = FBG(mmol/L)*fasting insulin(µIU/ml)/22.5. Thyroid ultrasound examination was conducted by trained sonographers using HivisionPreirus color doppler ultrasound diagnostic instrument in a 7.5 ~ 13.0mhz transducer (Hitachi, Japan). During the examination, all participants were kept in the supine position, with the neck extended and anterior cervical skin fully exposed. The length (a), width (b), thickness (c) of the two lobes of the thyroid of the participant were recorded. The volume of each lobe was calculated as (V = a*b*c*π/6).

### Diagnostic criteria of MetS

Overweight and obesity were diagnosed based on the standards issued by National Health and Family Planning Commission of the People’s Republic of China in 2004 [15]. MetS was diagnosed with 3 different criteria: children and teenagers MetS diagnostic criteria recommended by Pediatric Academy of Chinese Medical Association (Chinese criteria) [11], the International Diabetes Federation Criteria in 2007(IDF criteria) [12], and Cook Criteria released by the National Cholesterol Education Program-Adult Treatment Panel in 2003 (Cook criteria) [13]. The detection rates of components of MetS under three different diagnostic criteria were compared.

### Statistical analysis

Data were put into a double-track system using EpiData 3.1 soft-ware (EpiData Association, Odense, Denmark). Statistical analyses were performed using the SPSS 24 software package (SPSS, Inc., Chicago, IL, USA). The data were presented as mean ± standard deviation and categorical data was presented in number and percentage. Comparisons between groups were conducted with t-test or one-way analysis of variance. Linear regression and multiple linear regression were applied to examine the relation between thyroid volume and physical and biochemical parameters. The detection rates of MetS and its components under three different diagnostic criteria were evaluated by the *χ*^*2*^ -test. A two-tailed *P* value of < 0.05 was considered statistically significant.

## Results

Of the 1 097 adolescents in this study, 610 (55.6 %) were males and 487 (44.4 %) were female. The mean age was 13.05 ± 0.98 (range 12–15 years). In anthropometric and laboratory tests, systolic blood pressure, height, weight, waist circumference, hip circumference, fasting blood glucose and uric acid in the males were significantly higher than those in the females (*P* all < 0.01). However, diastolic blood pressure, heart rate, TC, TG, HDL-C, LDL-C, fasting insulin and BMI in the females were significantly higher than those in the males (*P* all < 0.01). Thyroid volume in the female is significantly larger than that of the male (*P* < 0.01) (Table 1).

**Table 1:** Basic characteristics of 1097 adolescents (mean ± SD)

gender	male	female	total
n (%)	610(55.6 %)	487(44.4 %)	1097
age (year)	13.03 ± 1.05	13.08 ± 0.90	13.05 ± 0.98
systolic blood pressure (mmHg)	117.44 ± 13.13**	112.47 ± 11.68	115.23 ± 12.74
diastolic blood pressure (mmHg)	70.63 ± 8.34**	72.86 ± 8.12	71.62 ± 8.31
heart rate (/min)	87.83 ± 11.93**	90.43 ± 12.53	88.98 ± 12.26
height (cm)	166.15 ± 9.70**	160.16 ± 8.31	163.50 ± 9.58
weight (Kg)	56.00 ± 13.37**	51.93 ± 10.92	54.20 ± 12.51
waist circumference (cm)	68.39 ± 10.18**	63.92 ± 6.82	66.41 ± 9.12
hip circumference (cm)	86.13 ± 8.74*	84.88 ± 7.70	85.58 ± 8.31
fasting blood glucose (mmol/L)	4.99 ± 0.39**	4.88 ± 0.35	4.94 ± 0.38
total Cholesterol (mmol/L)	4.00 ± 0.77**	4.29 ± 0.76	4.13 ± 0.78
triglyceride (mmol/L)	0.86 ± 0.48**	0.95 ± 0.44	0.90 ± 0.47
HDL-C (mmol/L)	1.54 ± 0.30**	1.64 ± 0.32	1.58 ± 0.31
LDL-C (mmol/L)	2.01 ± 0.61**	2.14 ± 0.62	2.07 ± 0.62
uric acid (umol/L)	411.91 ± 90.33**	340.47 ± 74.50	380.31 ± 90.88
fasting insulin (µIU/ml)	12.20 ± 7.47	12.97 ± 5.41	12.5 ± 4.64
HOMA-IR	2.74 ± 1.80**	2.83 ± 1.23	2.78 ± 1.58
BMI (Kg/m2)	20.32 ± 6.99	20.52 ± 7.79	20.41 ± 7.35
thyroid volume (mL)	5.40 ± 2.01**	5.82 ± 2.04	5.59 ± 2.03

*HDL-C* high-density lipoprotein cholesterol, *LDL-C* low-density lipoprotein cholesterol, *HOMA-IR* homeostasis model assessment of insulin resistance, *BMI* body mass index

*P < 0.05, **P < 0.01

We explored the relationship between the number of MetS components (under Chinese criterion) and thyroid volume with one-way ANOVA. With the increase of number of MetS components, thyroid volume of adolescents also increased significantly (*F* = 10.64, *P* < 0.01) (Table 3). When the number was 0, 1, 2, 3, the thyroid volume was 5.41 ± 1.61ml, 5.67 ± 2.16ml, 6.24 ± 3.27ml, 7.09 ± 3.81ml, respectively (Table 2).

**Table 2:** Association between the number of MetS components and thyroid volume

number of MetS components	n	thyroid volume(ml)(mean ± SD)	*F*	*P* value
0^a^	692	5.41 ± 1.61	10.64	< 0.01
1^b^	302	5.67 ± 2.16		
2^c^	70	6.24 ± 3.27		
3and more^d^	33	7.09 ± 3.81		

Multiple LSD-t test: a VS. b, *P* = 0.60; a VS. c, *P* = 0.001; a VS. d, *P *< 0.001; b VS. C, *P* = 0.033; b VS. d, *P *< 0.001; c VS. d, *P* = 0.043

In our study, the mean thyroid volume in different groups based on BMI were compared (Table 3). The thyroid volume in overweight group (*t* = 3.784, *P* < 0.001) and obese group (*t* = 5.068, *P* < 0.001) were significantly larger than that in normal group. The thyroid volume of obese females was significantly larger than that of normal females (*t* = 4.627, *P* < 0.001).

**Table 3:** Comparison of thyroid volume between normal, overweight and obese subjects of different genders (mean ± SD)

	All		Male		Female	
	n	thyroid volume(mL)	n	thyroid volume(mL)	n	thyroid volume(mL)
normal (*n* = 857)	459	5.39 ± 1.76	459	5.16 ± 1.66	398	5.66 ± 1.84
overweight (*n* = 152)	98	5.92 ± 1.79**	98	5.87 ± 1.91**	54	6.02 ± 1.58
obesity (*n* = 88)	53	6.89 ± 3.73**	53	6.60 ± 3.74**	35	7.33 ± 3.71**

VS. normal, *P < 0.05, **P < 0.01

**Fig. 1:**
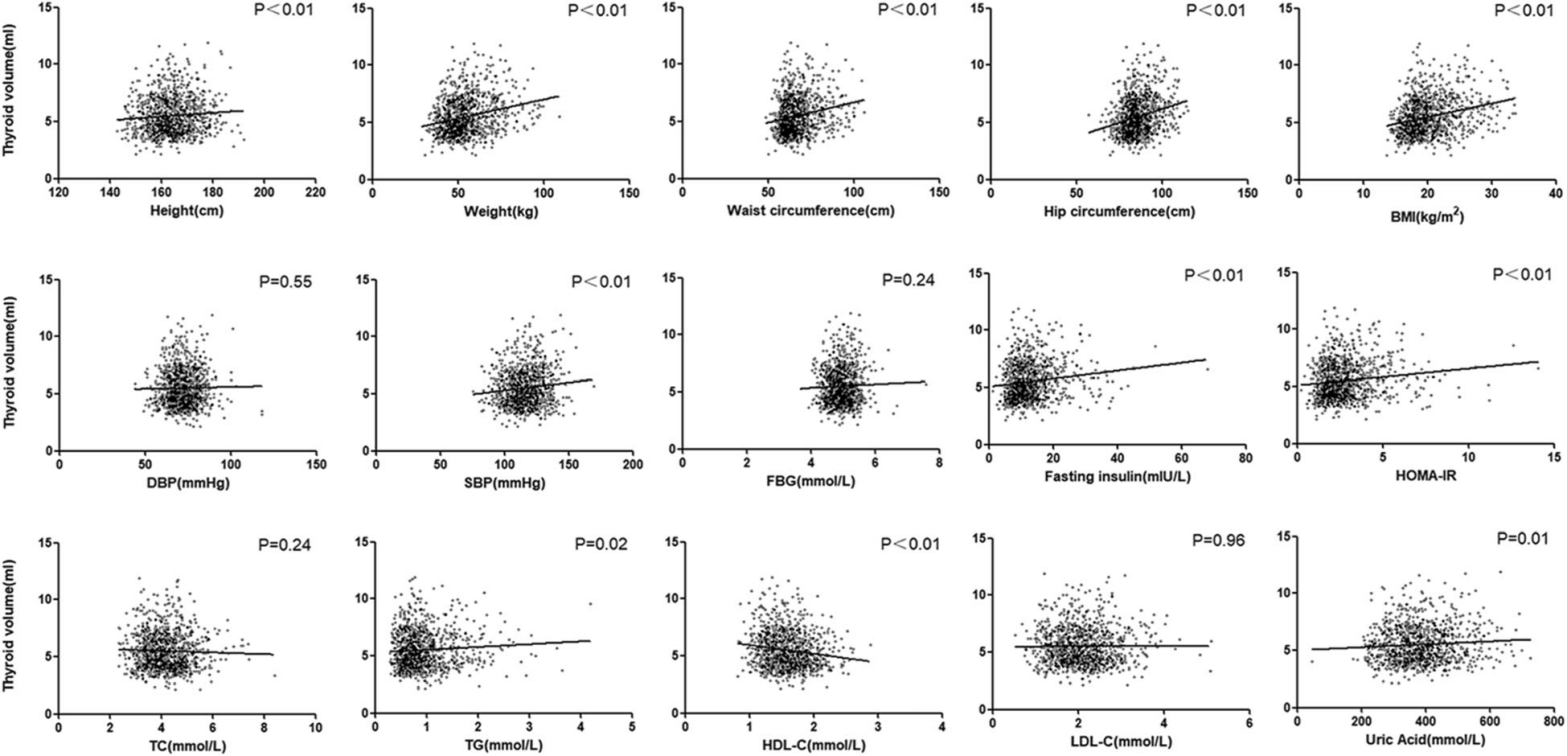
Linear Regression between physical and biochemical parameters and thyroid volume

Simple linear regression showed a significant positive association between height, weight, BMI, waist circumference, hip circumference, systolic blood pressure, fasting insulin, HOMA-IR, uric acid, triglycerides, and thyroid volume (all *P* < 0.001). Meanwhile, HDL-C was negatively associated with thyroid volume (*P* < 0.001) (Fig. 1).

Multiple linear regression is applied to predict the thyroid volume according to the metabolism related parameters. There is a linear relationship between independent variable and dependent variable after drawing partial regression plot and plot between studentized residuals and predicted value. By drawing the plot between the studentized residuals and the unstandardized predicted value, the variance of the residual is proved to be homogeneous. The regression tolerance was greater than 0.1, and variables with multicollinearity were not included in the regression model. In the outlier test, there were 13 observed values with studentized deleted residuals greater than 3 standard deviations, and they were deleted. The P-P diagram indicates that the residual is approximately normally distributed. The regression model was statistically significant (*F* = 11.93, *P* < 0.01, adjusted *R*^*2*^ = 0.178). The included variables with statistically significant effects on thyroid volume were sex (*β* = 0.607: 95 %CI: 0.406 ~ 0.808; *P* < 0.01), age (*β* = 0.200, 95 %CI: 0.107 ~ 0.294; *P* < 0.01), systolic blood pressure (*β* = 0.010, 95 %CI: 0.002 ~ 0.018; *P* < 0.01), waist circumference (*β* = 0.029, 95 %CI: 0.015 ~ 0.042; *P* < 0.01), and WHtR (*β* = 3.317, 95 %CI: 1.661 ~ 4.973; *P* < 0.01)(Table 4).

**Table 4:** Association between physical and biochemical parameters and thyroid volume

Parameters	β	95 %CI		*P* value
		lower	upper	
age	0.607	0.406	0.808	< 0.01
gender	0.200	0.107	0.294	< 0.01
DBP	0.000	-0.011	0.012	0.974
Heart rate	-0.007	-0.014	0.000	0.057
WC	0.029	0.015	0.042	< 0.01
FBG	0.101	-0.149	0.350	0.428
TG	0.096	-0.119	0.311	0.382
HDL-C	-0.073	-0.376	0.230	0.637
LDL-C	-0.077	-0.223	0.069	0.298
UA	0.000	-0.001	0.001	0.839
HOMA-IR	0.029	-0.043	0.100	0.430
WHtR	3.317	1.661	4.973	< 0.01

*DBP* diastolic blood pressure, *WC* waist circumference, *FBG* fasting blood glucose, *TG* triglyceride, *HDL-C* high-density lipoprotein cholesterol, *LDL-C* low-density lipoprotein cholesterol, *UA* uric acid, *HOMA-IR* homeostasis model assessment of insulin resistance, *WHtR* waist-to-height ratio

We also analyzed the association between thyroid volume and central obesity in adolescents. The volume was not found to be significantly larger in adolescents who suffered from central obesity along (*F* = 2.51, *P* = 0.057). Multiple LSD-t test find, however, thyroid volume in adolescents with central obesity complicated with hypertension was significantly larger than that of normal adolescents (*P* = 0.038) (Table 5).

**Table 5:** Comparison among groups in central obesity combined with other components

Number of MetS components	N	Thyroid volume (ml) (mean ± SD)	F	*P* value
Normal^a^	691	5.41 ± 1.61	2.51	0.057
Central obesity	37	5.59 ± 1.53		
Central obesity + hypertension^b^	28	5.96 ± 1.60		
Central obesity + dyslipidemia	14	6.04 ± 1.53		

Multiple LSD-t test: a VS. b, *P* = 0.038

The prevalence of MetS in children and adolescents in Changzhou was 2.3 %, 2.8 %, and 3.0 %, according to the Chinese criterion, IDF criterion, and Cook criterion, respectively, but there were no significant differences among 3 criteria (*P* = 0.549) (Table 6). The detection rates of hypertension by IDF criterion (*χ*^*2*^ = 22.179, *P* < 0.01) and Cook criterion (*χ*^*2*^ = 26.507, *P* < 0.01) were significantly higher than that by Chinese criterion. Chinese criterion achieved the lowest detection rate of triglyceride increase, exceeded by IDF criterion. Cook criterion brought with the highest detection rate (compared with Chinese standard (*χ*^*2*^ = 17,837, *P* < 0.01); compared with IDF criterion, *χ*^*2*^ = 13.028, *P* < 0.01). The three diagnostic criteria showed a consistent diagnostic value for obesity and hyperglycemia (Table 6).

**Table 6:** Prevalences of MetS and its component under three criteria [n(%)]

	Chinese criterion	IDF criterion	Cook criterion
Obesity	111(10.1 %)	111(10.1 %)	111(10.1 %)
Hypertension	113(10.3 %)a	189(17.2 %)	197(18.0 %)
Low HDL-C	21(1.9 %)	27(2.5 %)	27(2.5 %)
Hypertriglyceridemia	121(11.0 %)b	73(6.7 %)c	190(17.3 %)d
Hyperglycemia	35(3.2 %)	67(3.2 %)	35(3.2 %)
Metabolic syndrome	33(2.3 %)	31(2.8 %)	33(3.0 %)

Chinese criterion VS. IDF criterion, Cook criterion, ^a^*P *< 0.01; Comparison among 3 criterion, ^b,c,d^*P *< 0.01

## Discussion

In this study, obesity and the number of MetS components were significantly associated with larger thyroid volume. Waist circumference and WHtR were risk factors for thyroid enlargement. Meanwhile, different diagnostic criteria did not affect the detection rate of MetS.

Our data shows that the thyroid volume of female adolescents was significantly larger than that of male adolescents, which may be related to the fact that female adolescents enter puberty earlier than male adolescents. Previous studies have shown that estrogen promoted the proliferation of thyroid cells [16] and a few thyroid stem cells [17], a process that is thought to be associated with female’s goiter.

The association between MetS and thyroid gland volume has been verified in adults [7, 18, 19], but not in children and adolescents. Several guidelines have emphasized the importance of obesity in the diagnosis of MetS. Among them, the China criterion recommended central obesity as a necessary for diagnosis of MetS. We did not find a significant increase of thyroid volume in adolescents who suffered central obesity only. Instead, central obesity complicated with hypertension may associated with increased thyroid volume. Previous studies have found that subclinical hypothyroidism is associated to hypertension in adolescents [6]. At present, however, there is little evidence to prove the association between hypertension and thyroid volume. In this study, no significant difference in thyroid volume was observed between overweight and obese group; however, both had significantly larger thyroid volume than the normal group. It indicated that thyroid volume in adolescents is closely related to overweight and obesity. However, it may take a long time for obesity to exert its further effect on the formation of goiter.

The number of MetS components increased with thyroid volume in this study. Thyroid volume was the largest with all three components together. Interestingly, BMI, waist circumference, WHtR, systolic blood pressure, mean arterial pressure, fasting insulin, HOMA-IR, and triglycerides were all positively related to thyroid volume, while HDL-C was negatively related with thyroid volume. It is consistent with the conclusions of our previous clinical study [6] Waist circumference was found to be an independent risk factor for thyroid enlargement [18]. We have also found that central obesity can increase the risk of thyroid nodules [4]. In this study, there is no multicollinearity in waist circumference and WHtR. Multiple linear regression showed that waist circumference and WHtR were risk factors for goiter. Moreover, WHtR may be a more profound parameter. Overall, central obesity can be regarded as an independent risk factor of thyroid enlargement.

In adolescents, increased thyroid volume was positively associated with insulin level and HOMA-IR. Insulin resistance may be a major cause of MetS [20]. Insulin receptors are expressed in thyroid cells [21]. Excessive insulin can bind to its receptors expressed on thyroid cells, and activate AMPK-involved pathways, thereby leading to mitosis [22]. On the other hand, insulin also activates insulin growth factor (IGF)-1 receptors. A long-term insulin stimulation in thyroid follicular cells leads to cell proliferation and thus goiter [23]. IGF-1 also can increase the sensitivity of TSH, promote the proliferation of thyroid cells, and increase the colloidal volume, which can partially explain the pathogenesis of thyroid enlargement [24–26]. In adults, insulin resistance has been found to be positively associated with the incidence of goiter and thyroid tumors [25–27].

The detection rate varies with diagnostic criterion in children and adolescents with MetS, ranging from 0.2 to 38.9 % [14, 28]. In a systematic review including 85 pediatric studies, the median prevalence of MetS in the whole population is 3.3 % and still on the rise [28]. In our study, the detection rates of MetS in children and adolescents in Changzhou was 2.3 %, 2.8 %, and 3.0 %, according to the Chinese criterion, IDF criterion, and Cook criterion, respectively. All showed no statistical difference. It was reported that the prevalence of MetS in north American adolescents and children was 9.6 % under IDF criterion [29], more than three times of that in eastern China. However, each criterion showed a significant difference in detecting MetS components, most obviously in hypertension and triglyceride elevation. The main reason was that the cut-off values set for these three components were significantly distinctive among the three criteria.

There are still some limitations in this study. Thyroid function indexes, especially serum TSH levels, was not measured in adolescents. Diabetes, obesity, and elevated lipid levels were all associated with serum thyrotropin levels [30, 31]. The detection of serum TSH levels in adolescents may provide more insights to understand the mutual effects between metabolic disorders and thyroid abnormalities.

In conclusion, this study showed that overweight, obesity and number of metabolic syndrome components are significantly associated with thyroid volume in adolescents. There were no differences of detection rate of MetS in adolescents under different diagnostic criteria.

## Data Availability

The datasets used and/or analyzed during the current study are available from the corresponding author on reasonable request.
